# Olfactive stimulation interventions for managing procedural pain in preterm and full-term neonates: a systematic review protocol

**DOI:** 10.1186/s13643-017-0589-1

**Published:** 2017-10-17

**Authors:** Gwenaëlle De Clifford-Faugère, Andréane Lavallée, Marilyn Aita

**Affiliations:** 1 Faculty of Nursing, Université de Montréal, C.P. 6128 Succ. Centre-ville, Montreal, H3C 3J7 Canada; 2 CHU Sainte-Justine Research Center, Montreal, Canada; 3 Quebec Network of Nursing Intervention Research (RRISIQ), Montreal, Canada

**Keywords:** Pain, Odors, Non-pharmacological intervention, Preterm, Full-term neonate, Systematic review

## Abstract

**Background:**

While hospitalized in the NICU, preterm neonates undergo many painful procedures. This may be the same for full-term neonates when longer hospitalization is required. Untreated and repeated pain has short-term as well as long-term consequences for these neonates. Pharmacological pain management methods have many limitations in their applications for both preterm and full-term neonates. A combination of different non-pharmacological methods is recommended for pain management. The effect of olfactive stimulation as a non-pharmacological pain management method was investigated by a few studies in the past years with premature and term neonates, but no systematic review has been conducted. The objective of this systematic review is to evaluate the effect of olfactive stimulation intervention on the pain response of preterm and full-term neonates during painful procedures.

**Methods:**

An electronic search will be conducted in various databases such as PubMed (1946 to date), MEDLINE (1946 to date), CINAHL (1981 to date), Embase (1947 to date), PsycINFO (1806 to date), Web of Science (1945 to date), CENTRAL and Scopus (1960 to date), and Proquest, without restriction for the year of publication. Only studies published in English or French will be included. The search will be conducted using the following three concepts: pain, odors, and neonates. Selection of articles, data extraction, and assessment of risk of bias will be conducted by two independent researchers. A third researcher will intervene in case of disagreement. According to the availability of studies and data homogeneity, the results will be combined to perform a meta-analysis, or they will be described by a narrative synthesis.

**Discussion:**

This systematic review will provide light on the current state of knowledge on the effectiveness of olfactive stimulation interventions for managing pain in preterm and full-term neonates. This review will guide clinical practice as well as research to improve preterm and full-term neonates’ pain management and prevent short-term and long-term complications caused by pain.

**Systematic review registration:**

PROSPERO CRD42017058021

**Electronic supplementary material:**

The online version of this article (10.1186/s13643-017-0589-1) contains supplementary material, which is available to authorized users.

## Background

It has been known, for several decades, that neonates feel pain [1]. Almost 8% of births are premature in North America [2]. Full-term and preterm neonates are subject to painful procedures during their hospitalization in the neonatal unit. These procedures are quite frequent, varying from 7.5 to 17.3 per week, depending on the unit and the neonate’s health status [3]. The preterm neonates are especially vulnerable to pain [4] due to the immaturity of their neurological system. According to a study conducted in 2011 by Johnston, Barrington [5], the frequency of painful procedures experienced by preterm neonates reach 33 per week during their hospitalization in the neonatal unit. For full-term and preterm neonates, only half of these painful procedures are accompanied by a pharmacological or non-pharmacological pain management intervention [3, 5, 6]. Moreover, untreated pain has short-term consequences such as physiological and behavioral changes in neonates [4, 7]. Furthermore, untreated and repeated pain could cause sensory disorders associated with the number of painful procedures received during the hospitalization, for full-term and preterm neonates [8, 9], as well as lower mental and psychomotor indices of development in preterm neonates [10]. So, to improve health outcomes, it appears essential to effectively relieve pain in preterm and term neonates. This paper presents the protocol for a systematic review considering the effectiveness of olfactive stimulation interventions as a pain management intervention during the ten most frequent painful procedures [6] to which full-term and preterm neonates are exposed throughout neonatal hospitalization.

Despite advances in this area of knowledge, there is still little evidence supporting pain management interventions for full-term and preterm neonates [11]. A lack of knowledge still persists about the long-term impact of pharmacological pain management methods, limiting their application [12]. Systematic reviews have found that effective non-pharmacological interventions for full-term neonates are sucrose administration, which could be combined to non-nutritive sucking [13, 14], breastfeeding, and skin-to-skin contact [15]. For preterm neonates, only sucrose, with or without non-nutritive sucking [13], or skin-to-skin contact [15] is considered to be effective interventions. However, these interventions are not always applicable depending on the circumstances as breastfeeding requires the mothers’ presence, and skin-to-skin contact requires one of the two parents. In addition, it is recommended by the American Academy of Pediatrics [16] to combine several non-pharmacological methods in order to effectively relieve pain in preterm and full-term neonates. Olfactive stimulation interventions could be easily combined with other interventions as it uses the olfactive sphere which is little stimulated by the above cited interventions.

### How the intervention might work

During embryogenesis, the olfactory system is growing rapidly since as early as 11 weeks of gestation, the first functional cells are present [17, 18]. Full-term and preterm neonates have acquired the necessary skills allowing them to detect, distinguish, and recognize a smell as soon as 28 weeks of gestation [17]. Furthermore, they may express preferences when they are exposed to different smells [19]. For neonates, olfactive stimulation interventions seem to be particularly interesting to investigate. To our knowledge, the effectiveness of this type of intervention has never been evaluated by a systematic review.

Several other elements, let us hypothesize that an olfactive intervention could contribute to pain management in neonates. The skin-to-skin contact where the neonate smells his own mother’s odor helps decrease pain [15]. Breastfeeding is also an effective intervention for neonates, where they can smell their mother’s milk [14]. Olfactive stimulation intervention could also act as a distraction. By linking pain with olfaction physiology, we hypothesize that olfactive stimulation could have an impact on the emotional and affective pain components.

### Importance of doing the review

To our knowledge, no systematic review has evaluated the effectiveness of olfactive stimulation interventions on full-term and preterm neonates’ pain response. Considering short-term and long-term consequences of repeated and untreated pain, as well as the lack of knowledge about the other pain management interventions, it is essential to evaluate the effectiveness of this type of intervention. If possible, we will also evaluate the combined effect of olfactive stimulation intervention with another pain management intervention, in accordance with the recommendations of the American Academy of Pediatrics [16]. A better knowledge of effective pain management interventions for neonates will guide the clinical practice and future research. The purpose of this systematic review is to assess the effectiveness of olfactive stimulation interventions on full-term and preterm neonates’ pain response.

### Objective

The objective of this systematic review is to answer the following question: What is the effectiveness of olfactive stimulation interventions on full-term and preterm neonates’ pain response during a painful procedure compared to standard of care?

## Methods

We followed the Preferred Reporting Items for Systematic review and Meta-Analysis Protocols (PRISMA-P) recommendations for the development of this protocol [see Additional file 1] [20].

### Eligibility criteria

#### Types of studies

Randomized controlled trials will be included in the systematic review. Quasi-experimental studies will only be included in the descriptive synthesis and excluded from the meta-analysis of this review. There will be no restrictions in regard to the clinical settings where the olfactive stimulation interventions have been evaluated.

#### Types of participants

Participants will be full-term neonates (> 37 weeks of gestation) and preterm neonates (< than 37 weeks of gestation) undergoing a painful procedure. The ten most frequent painful procedures in full-term and preterm neonates documented will be considered in this review [6]: (1) nasal aspiration, (2) tracheal aspiration, (3) heel prick, (4) adhesive removal, (5) gastric tube insertion, (6) venipuncture, (7) arterial puncture, (8) installation of peripheral intravenous cannula, (9) chest physiotherapy, and (10) removal of peripheral intravenous line.

#### Types of interventions

The olfactive stimulation interventions that will be included in this systematic review include all types of odors that neonates may have been exposed to during the intervention, whether natural (e.g., milk) or artificial (e.g., vanilla). All methods of odor administration to the neonates will be considered (e.g., gauze, cotton). Studies examining the effectiveness of multisensory interventions will not be considered in this systematic review, because it will not be possible to isolate the effect of the olfactive stimulation intervention on pain response.

#### Comparators

Comparators include placebo, standard of care, or different odors. Standard care can be defined as any care or intervention carried out in the clinical setting in order to manage procedural pain in neonates. Standard care must have been received by the two groups in the selected studies. For ethical considerations, the control group may be an active group, where the evaluation will focus on the combined effect of the olfactive stimulation intervention with, for example, sucrose or swaddling (considered in some areas as standard care) compared to sucrose alone or to the swaddling alone.

#### Primary outcome

Full-term and preterm neonates’ pain response is the primary outcome of this systematic review. Pain has different components that evaluate, among other things, behavioral and physiological indicators [12]. Pain response could be measured by behavioral indicators (i.e., crying, facial reactions), physiological indicators (i.e., cortisol levels, heart rate, respiratory rate, oxygen saturation), and contextual indicators (i.e., gestational age). Procedural pain assessment tools are different for full-term and preterm neonates. Pain response should be measured in studies by valid and reliable scales for the two populations in order to be included in the meta-analysis of this review, such as the premature infant pain profile (PIPP) adapted to measure pain in neonates from 28 to 40 weeks of gestation (WG), the neonatal infant pain scale (NIPS) from 26 to 47 WG, the neonatal facial coding system (NFCS) from 26 to 47 WG, and the *Douleur aigue du nouveau-né* [acute pain of the newborn] (DAN) from 24 to 41 WG.

#### Secondary outcome

Adverse events of olfactive stimulation interventions will be considered as a secondary outcome. Secondary outcome will only be considered if the study’s primary outcome is pain management. Adverse events will be evaluated as follows: (1) adverse events reported by authors, (2) none adverse events reported by authors, and (3) adverse events not mention by authors.

### Information sources

#### Search strategy

A search by Mesh terms and keywords will be performed with the three central concepts: pain, odors, and neonates (term and preterm). The search strategy has been designed in collaboration with an experienced librarian using these three concepts [see Additional file 2]. The research strategy will be adapted to each specific database. No year of publication restrictions will be applied because olfactive stimulation interventions have never been evaluated by a systematic review. Only studies published in English or French will be considered in this review.

#### Electronic databases

Research will be conducted in different electronic databases:

- PubMed, 1946 to date
- MEDLINE (OVID), 1946 to date
- CINAHL (EBSCOHost), 1981 to date
- EMBASE (OVID), 1947 to date
- PsycINFO (OVID), 1806 to date
- Web of Science, 1945 to date
- Cochrane Central Register of Controlled Trials (CENTRAL), The Cochrane Library
- Scopus, 1960 to date
- ProQuest Dissertation and Theses Global British Nursing Index

#### Searching other resources

Journals focused on neonatology, neonates, and pain will be consulted. Conference abstracts will be searched (BIOSIS, Biological abstract). References of the included studies will be screened for other potential studies. If data is missing, the authors will be contacted to provide additional data, as well as to identify unpublished studies. We will also seek the ClinicalTrials.gov website, the World Health Organization International Clinical Trials Registry Platform, and metaRegister of controlled trials for ongoing or unpublished randomized clinical trials.

### Data collection and analysis

#### Studies selection

The process of study selection will be illustrated by a PRISMA diagram [21] and will be made according to the steps described by Pai et al. [22]. Duplicates of studies will first be eliminated from the studies identified through the database searches. Study references will be saved in a bibliographic management software (EndNote© X7). Study selection will be performed in two steps. The first step will be a selection of studies from their titles and abstracts, by two independent researchers. In case of disagreement, consensus will be sought by discussion, and if necessary, a third expert intervention will be sought. In the second step, full studies selected previously will be read by two independent researchers in order to decide on the inclusion or exclusion of each study according to our selection criteria. Additional data could be requested to the study authors, if necessary to make a decision about its inclusion in the systematic review. Disagreements will be solved by consensus or by a third expert if necessary. Reasons for study exclusion will be documented. Each included study will have a unique identification number.

#### Data extraction and management

Two independent researchers will extract the data of ten studies and then compared to ensure consistency between the two researchers. A third researcher will be requested in case of disagreement. For each study, extracted data will be:

- Characteristics of the study: authors, year, country of publication, title of the study, and trial number;
- Methodology: design, purpose of the study, type of randomization, control group, sample size, study setting, method of data collection, and instruments used (i.e., video.);
- Participants: sociodemographic characteristics (gestational age at birth, age at the time of the intervention, birth weight, breastfeeding, mode of delivery, respiratory support during the intervention) and exclusion and inclusion criteria;
- Painful procedure: painful procedure type, frequency, and duration;
- Intervention: description (duration, type of odors, exposure time to the odors, frequency, distance of the odor to the neonates’ head, comparator); and
- Results of the study.

These data will be extracted for each study and entered into a data extraction form. Then, data will be entered in the software review manager (RevMan 5.1). If several papers of the same study are found, the paper containing the most comprehensive information will be included. Others will be excluded, unless they report additional data that are not exactly the same and that are also related to pain or pain components, in which case they will also be included.

#### Dealing with missing data

When data are missing in studies, authors will be contacted. If data cannot be obtained, an imputation method will be used [23] and then a sensitivity analysis will be performed to assess the impact of imputing data in the studies’ data synthesis. Otherwise, a descriptive synthesis will be carried out.

#### Assessment of risk of bias

Risk of bias of each selected study will be assessed by two independent researchers. In case of disagreement, a third expert will be consulted. We will use the Cochrane Risk of Bias Assessment Tool [24] to assess the risk of bias according to the following items: (1) random sequence generation (selection bias); (2) allocation concealment (selection bias); (3) blinding of participants, staff, and outcome assessment (detection and performance bias); (4) incomplete outcome data (attrition bias); (5) selective outcome reporting (presence reporting bias); (6) other sources of bias (other bias has not been addressed in other sections); and (7) overall risk of bias. Authors of included studies will be contacted in case of doubts during the evaluation of a bias. Each of these biases will be classified in terms of uncertain risk, low risk, and high risk. In the review, this information will be presented in a table done by the RevMan 5.1 software. Risk of bias will be taken in consideration for the analysis and the interpretation of the results.

#### Data synthesis

A descriptive synthesis will summarize data from different studies. Furthermore, a meta-analysis will be conducted according to the number of included studies and their data homogeneity. For continuous data, we will report the average as well as the mean difference (MD) for outcomes measured by the same scale and standardized mean difference (SMD) for outcomes measured by different scales, both with a 95% confidence interval. For dichotomous data, we will use a risk ratio with a 95% confidence interval. Heterogeneity will be statistically verified by a chi-square test, with a *p* value < 0.1 for statistical significance, as well as report the *I* ^2^. *I* ^2^ will be interpreted as the following [24]: 0–40%: heterogeneity might not be important, 30–60%: may represent moderate heterogeneity, 50–90%: may represent substantial heterogeneity, and 75–100%: considerable heterogeneity. If a *I* ^2^ > 50% is found, a subgroup analysis will be carried out in order to try to explain it. The RevMan 5.1 software will be used to combine studies. First, we will choose a fixed effect model; then, we will change to the random effect model if the data are not sufficiently homogenous. Otherwise, a descriptive synthesis, taking into account the design, the randomization, the intervention, and how pain response was measured, as well as the results, will be conducted [24].

#### Subgroup analysis

If possible, full-term and preterm neonates will be separated into subgroups for analysis. Moreover, if possible, subgroup analysis will be conducted by considering different types of odors used in studies and the different pain components in the instruments (i.e., behavioral, physiological).

#### Publication bias

If more than ten studies meet the inclusion criteria, publication bias will be assessed graphically through a Funnel plot done by the RevMan software as well as statistically by a regression of Egger test [24].

#### Quality of evidence

Assessment of data quality will be performed by the Grading of Recommendations Assessment, Development and Evaluation working group methodology (GRADE), including five considerations: limitations, effect consistency, imprecision, indirectness, and publication bias [25]. Depending on the GRADE score, quality of the evidence will be ranked high, medium, low, or very low, by two independent experts and will be taken into account in the recommendations based on data analysis. In addition, studies may be excluded based on the quality of evidence.

#### Discussion

Given consequences of repeated and untreated pain, it is important to evaluate the effectiveness of interventions for pain management in preterm and full-term neonates during painful procedures. This systematic review will contribute to the advancement of knowledge on pain management in preterm and full-term neonates to guide clinical practice and research.

## Additional files

Additional file 1: PRISMA-P 2015 checklist (DOCX 29 kb)

Additional file 2: PubMed search strategy. (DOCX 13 kb)

## Abbreviations

DAN: *Douleur aigue du nouveau-né*
GRADE: Grading of recommendations assessment, development, and evaluation working group methodology
NFCS: Neonatal facial coding system
NICU: Neonatal intensive care unit
NIPS: Neonatal infant pain scale
PIPP: Premature infant pain profile
PRISMA-P: Preferred reporting items for systematic review and meta-analysis protocols
WG: Weeks of gestation
