# Supplementary material for: Olfactive stimulation interventions for managing procedural pain in preterm and full-term neonates: a systematic review protocol
Source: Syst Rev. 2017 Oct 17;6:203. doi: 10.1186/s13643-017-0589-1 (PMC5646110; doi:10.1186/s13643-017-0589-1)
Supplement: Supplementary file 2 — PubMed search strategy. (DOCX 13 kb) [file 13643_2017_589_MOESM2_ESM.docx]

Additional file 2

PubMed search strategy

|  | **Équation de recherche** | **Résultats** |
| --- | --- | --- |
| 1 | "Odorants"[Mesh] OR "Oils, Volatile"[Mesh] OR "Smell"[Mesh] OR "Perfume"[Mesh] OR "Flower Essences"[Mesh] OR "Aromatherapy"[Mesh] OR "Olfactory Perception"[Mesh] | 37 859 |
| 2 | [Title/Abstract] = (odor* OR smell* OR scent* OR fragranc* OR olfact* OR perfume* OR aroma OR aromatherap* OR "essential oil" OR "essential oils" OR redolence* OR incense*) | 79 183 |
| 3 | "Pain"[Mesh] OR "Pain Management"[Mesh] OR "Pain Perception"[Mesh] OR "Pain Threshold"[Mesh] OR "Pain Measurement"[Mesh] OR "Stress, Physiological"[Mesh] OR "Crying"[Mesh] | 540 711 |
| 4 | [Title/Abstract] = (pain* OR cry OR crying OR cries OR scream* OR suffer* OR tear* OR grimac* OR agitat* OR distress OR stress* OR sooth* OR calm* OR sob* OR weep* OR ache* OR aching OR agony OR agon* OR afflict* OR anguish* OR cramp* OR discomfort OR irritat* OR sore* OR torment OR twinge*) | 1 854 884 |
| 5 | "Infant"[Mesh] OR "Neonatal Nursing"[Mesh] OR "Intensive Care, Neonatal"[Mesh] OR "Intensive Care Units, Neonatal"[Mesh] OR "Neonatology"[Mesh] OR "Neonatologists"[Mesh] | 1 026 963 |
| 6 | [Title/Abstract] = (neonat* OR baby OR babies OR newborn* OR infant* OR (child* AND (premature* OR preterm OR newborn* OR neonat*))) | 658 275 |
| 7 | (#1 OR #2) AND (#3 OR #4) AND (#5 OR #6) | 370 |
| 8 | #7 AND (English[Language] OR French[Language]) | 353 |
